# Hospital Meetings, &c.

**Published:** 1900-02-17

**Authors:** 


### HOSPITAL MEETINGS, &C.

### ST. MARK'S HOSPITAL.

The sixty-fourth annual meeting of St. Mark's Hospital was held at the Mansion House on Thursday, 8th instant, Mr. R. B. Martin, M.P., the treasurer, presiding.

The report presented showed that there had been during the past year an increase in both in-patients and out-patients. Mr. Alfred Cooper, who last year resigned his position as honorary surgeon, was appointed a consulting surgeon to the hospital. The accounts showed a total ordinary income of £1,900, and extraordinary income £2,874 — the latter consisting of £1,129, the balance of amount received in connection with the festival dinner in 1898; the remainder being legacies. The total expenditure amounted to £4,019. £156 was received from the Hospital Sunday Fund and £43 from the Hospital Saturday Fund; but the committee expressed their regret that that year again no part of the Prince of Wales's Fund had been allotted to them, though other hospitals whose needs they considered no greater had received grants; and went on to point out that it was only by careful management and the employment of legacies received during the year—which in their opinion ought to be treated as capital—to supplement its income, that the hospital had been able to meet its expenditure.

The Chairman, in moving the adoption of the report, emphasised the hospital's need of funds and the disappointment felt by the committee of management that they had again been left out by the Prince of Wales's Fund, which, he said, affected them both directly and indirectly, since many of their former subscribers now paid into that fund instead of to them direct. He said that the Sunday Fund, of which he was a member, went on a systematic plan in their distribution; but complained that the Prince of Wales's committee discriminated between one class of hospital and another. He considered that the fact of the establishment of a hospital and its support by the public was sufficient justification for its existence and claim on such a fund. He felt very strongly that a charity should not get into debt; and the fact that they had not done so was no reason why they should not participate in benefits in which others shared.

Other speakers expressed themselves in a similar sense, and, on the motion of Mr. Merriman, the following resolution was adopted: "That this meeting of governors of St. Mark's Hospital wish to place on record their feelings of deep disappointment and concern that this hospital has, again this year, not only been deprived of any participation in the Prince of Wales's Fund, but that the committee of such Fund should have seen fit to publish in their report a statement that they consider the financial position of this hospital does not seem to require help this year, the fact being that the hospital is urgently in need of funds, and has only been able to meet the year's expenditure by the employment of legacies received during the year, amounting to upwards of £570, and the balance of the amount derived from the festival dinner in 1898, and, therefore, by publishing their opinion as above, the aforesaid committee has done a serious injustice to the hospital, and lessened its chance of obtaining in the future the increased assistance it so sorely needs." It was also resolved to send a copy of this resolution to the secretary of the Prince of Wales's Fund, Mr. A. G. Sandeman promising if possible to bring the matter to the notice of the meeting of the Fund in the following week, when he hoped to be present.

The two retiring members of the committee—Mr. J. C. F. Tower and Mr. H. W. Whitbread—were re-elected, votes of thanks to the medical staff, the Ladies' Committee, and the honorary officers were adopted, and similar compliments to the Lord Mayor for the use of the Mansion House and to the chairman for presiding closed the proceedings.

